# Genetic Characterization of Highly Pathogenic Avian Influenza A(H5N8) Virus in Pakistani Live Bird Markets Reveals Rapid Diversification of Clade 2.3.4.4b Viruses

**DOI:** 10.3390/v13081633

**Published:** 2021-08-18

**Authors:** Muzaffar Ali, Tahir Yaqub, Muhammad Furqan Shahid, Foong Ying Wong, Nadia Mukhtar, Muhammad Naeem, Pauline Lam, Jayanthi Jayakumar, Gavin J. D. Smith, Yvonne C. F. Su

**Affiliations:** 1 Programme in Emerging Infectious Diseases, Duke-NUS Medical School, 8 College Road, Singapore 169857, Singapore; muzaffar.ali@uvas.edu.pk (M.A.); foongying.wong@duke-nus.edu.sg (F.Y.W.); pauline.lam@duke-nus.edu.sg (P.L.); jayanthi.jayakumar@duke-nus.edu.sg (J.J.); gavin.smith@duke-nus.edu.sg (G.J.D.S.); 2 Institute of Microbiology, University of Veterinary and Animal Sciences, Lahore 54600, Pakistan; tahiryaqub@uvas.edu.pk (T.Y.); furqan.shahid@uvas.edu.pk (M.F.S.); nadiamukhtar84@yahoo.com (N.M.); 3 Institute of Pure and Applied Biology, Bahauddin Zakariya University, Multan 60800, Pakistan; dr_naeembzu@yahoo.com; 4 SingHealth Duke-NUS Global Health Institute, SingHealth Duke-NUS Academic Medical Centre, Singapore 169857, Singapore; 5 Duke Global Health Institute, Duke University, Durham, NC 27710, USA

**Keywords:** evolution, poultry, zoonotic, influenza virus, molecular dating

## Abstract

The highly pathogenic (HPAI) avian influenza A(H5N1) viruses have undergone reassortment with multiple non-N1-subtype neuraminidase genes since 2008, leading to the emergence of H5Nx viruses. H5Nx viruses established themselves quickly in birds and disseminated from China to Africa, the Middle East, Europe and North America. Multiple genetic clades have successively evolved through frequent mutations and reassortment, posing a continuous threat to domestic poultry and causing substantial economic losses. Live bird markets are recognized as major sources of avian-to-human infection and for the emergence of zoonotic influenza. In Pakistan, the A(H5N1) virus was first reported in domestic birds in 2007; however, avian influenza surveillance is limited and there is a lack of knowledge on the evolution and transmission of the A(H5) virus in the country. We collected oropharyngeal swabs from domestic poultry and environmental samples from six different live bird markets during 2018–2019. We detected and sequenced HPAI A(H5N8) viruses from two chickens, one quail and one environmental sample in two markets. Temporal phylogenetics indicated that all novel HPAI A(H5N8) viruses belonged to clade 2.3.4.4b, with all eight genes of Pakistan A(H5N8) viruses most closely related to 2017 Saudi Arabia A(H5N8) viruses, which were likely introduced via cross-border transmission from neighboring regions approximately three months prior to virus detection into domestic poultry. Our data further revealed that clade 2.3.4.4b viruses underwent rapid lineage expansion in 2017 and acquired significant amino acid mutations, including mutations associated with increased haemagglutinin affinity to human α-2,6 receptors, prior to the first human A(H5N8) infection in Russian poultry workers in 2020. These results highlight the need for systematic avian influenza surveillance in live bird markets in Pakistan to monitor for potential A(H5Nx) variants that may arise from poultry populations.

## 1. Introduction

The highly pathogenic (HPAI) avian influenza A(H5N1) A/goose/Guangdong/1/1996 (Gs/Gd) virus emerged in China in 1996 and subsequently evolved into multiple genetic clades [1], causing widespread outbreaks in poultry, with millions of birds culled and intermittent human infection [2,3,4]. From 2008 onwards, extensive diversification of clade 2.3.4 A(H5N1) viruses has led to a prominent switch of NA-N1 to non-N1 subtype, resulting in the emergence and circulation of novel H5Nx reassortant viruses, including H5N2, H5N5, H5N6 and H5N8 [1,5]. Clade 2.3.4.4 HPAI A(H5N8) viruses have rapidly spread across many countries in Asia, Africa, Europe and the Middle East, leading to regional outbreaks with high mortality in wild and domestic birds [6,7,8]. Clade 2.3.4.4 HPAI A(H5N8) has further differentiated into at least 8 distinct phylogenetic groups, designated as clade 2.3.4.4a to clade 2.3.4.4h, which co-circulate in domestic and wild birds [1,9]. Of these, clade 2.3.4.4b viruses have received increased attention since the first human zoonotic infection with A(H5N8) virus was detected in poultry workers from Russia in December 2020 [10], reinforcing the risk of zoonotic transmission from poultry. Clade 2.3.4.4b H5N8 viruses were first detected in domestic ducks in eastern China in 2013 [11], which then subsequently spread to East Asia and Europe and eventually to North America from 2014 onwards [12,13]. Influenza A(H5) candidate vaccine strains are only available for three groups of clade 2.3.4.4 viruses, namely 2.3.4.4a (A/Sichuan/26221/2014 (H5N6)), 2.3.4.4c (A/gyrfalcon/Washington/41088-6-2014 (H5N8)) and 2.3.4.4e (A/duck/Hyogo/1/2016 (H5N6)), although A/Fujian-Sanyuan/21099/2017 (H5N6) has been selected as the candidate vaccine strain for clade 2.3.4.4b viruses [9].

Previous studies have shown that live bird markets (LBMs) and poultry trade are major contributors to the persistence and spatial distribution of avian influenza viruses (AIVs), particularly A(H5N1), A(H5N6), A(H7N9) and A(H9N2) subtype viruses [14,15,16,17]. The ability of AIVs to reassort among and between different subtypes facilitates the generation of novel viruses and is associated with sporadic human infections. According to the World Health Organization, 862 cases of human infection with A(H5N1) and 1568 human cases of A(H7N9) infection have been reported since 2013 [18]. More recently, the first human case with a low pathogenic avian influenza A(H10N3) reassortant virus was detected in May 2021 in Zhenjian City, China [19]. Surveillance for AIV in LBMs is routinely conducted in several Southeast Asian countries, including Cambodia, China, Bangladesh and Vietnam [17,20,21,22,23,24,25,26], revealing subtype prevalence and distribution.

Surveillance studies in Pakistan have frequently described the prevalence and evolutionary relationships of avian A(H9N2) viruses since 1990s, as the virus is endemically circulating in the poultry population [27,28,29,30,31]; however, avian influenza A(H5) virus is not well understood within the country. In 2006, HPAI A(H5N1) virus was first reported in commercial layer and breeder farms and was associated with subsequent outbreaks in domestic poultry in 2007–2008 [32,33]. The HA cleavage motifs of early avian Pakistan H5 virus (clade 2.2) contained the multiple basic amino acids (QGERRRKKR/GLF), which is characteristic of HPAI H5 virus [32,33]. This A(H5N1) virus has also affected a range of domesticated bird species (including crows, peacocks, turkeys and geese) in Pakistan [33], with 4 human A(H5N1) laboratory confirmed cases reported in 2007 [34]. Non-N1 H5Nx viruses have not been reported in Pakistan, where systematic AIV surveillance in live bird markets (LBMs) is lacking. In this study, we conducted AIV surveillance in Pakistani LBMs and collected oropharyngeal swabs from domestic birds, as well as environmental samples, in order to determine whether H5Nx viruses were present.

## 2. Materials and Methods

### 2.1. Surveillance and Sample Collection

**Figure 1:**
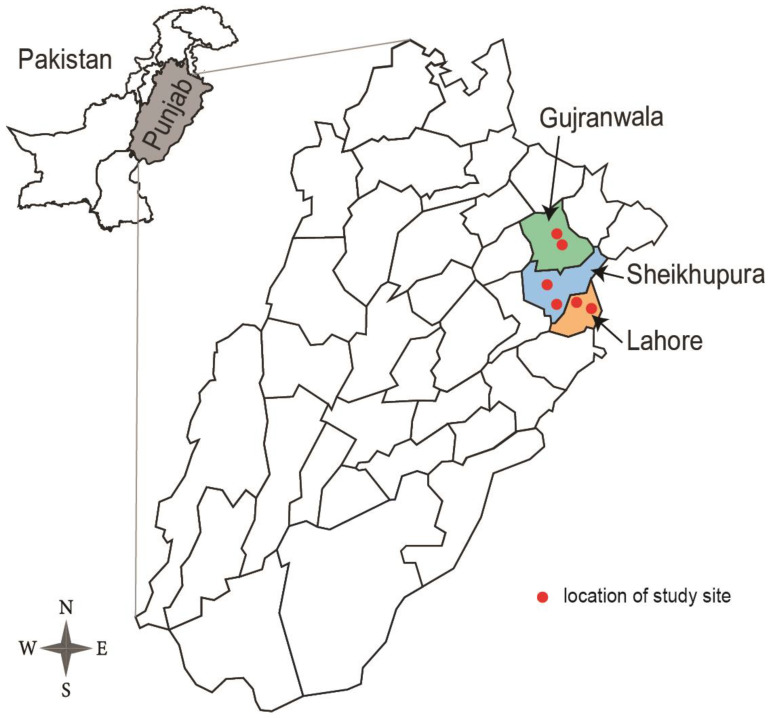
Geographical map of Punjab in Pakistan. Colored areas show the locations of the three districts included in this study. Red dots represent the locations of sampled live bird markets.

During September 2018–March 2019, avian influenza virus (AIV) surveillance was conducted in six LBMs in three districts (Gujranwala, Lahore and Sheikhupura) of Punjab Province, Pakistan (Figure 1). These LBMs sell fresh domesticated poultry meat and eggs for consumption. A wide range of bird species are housed, such as backyard and commercially farmed chickens, quails, ducks and pigeons. For each LBM, sample collections were conducted once a month. We collected 449 oropharyngeal swabs from domestic poultry (including chicken and quail) and 696 environmental samples (including chopping board surface, cages, cage drinking water, weighing scale, sewage water and water used for meat processing). All procedures were approved by the Ethical Review Committee at the University of Veterinary and Animal Sciences Lahore in Pakistan (DR/381). Pooled samples (up to *n* = 5) were cultured in 9-day-old embryonated chicken eggs for 48 h and amniotic–allantoic fluids were tested for influenza A virus by hemagglutination assay (HA) using 1% chicken red blood cells [35]. Individual samples from positive pools were subsequently cultured and tested by hemagglutination inhibition (HI) assay for the presence of H9 virus [28]. Hemagglutination-assay-positive but HI-assay-negative samples were then directly submitted for full genome sequencing to identify non-H9 viruses.

### 2.2. RNA Extraction and Next Generation Sequencing

Total viral RNA was extracted from the cultured isolates using the QIAamp Viral RNA Mini Kit (Qiagen, Hilden, Germany) following the manufacturer’s instructions. Full-genome sequencing of influenza A virus was performed by next-generation sequencing. Briefly, cDNA synthesis and RT-PCR amplification were performed using the SuperScript III Platinum One-step qRT-PCR kit (Thermo Fisher Scientific, Waltham, MA, USA) using the following three primers as previously described [36]: Opti1-F1-5′ GTTACGCGCCAGCAAAAGCAGG; Opti1-F2-5′ GTTACGCGCCAGCGAAAGCAGG; Opti1-R1-5′ GTTACGCGCCAGTAGAAACAAGG (Integrated DNA Technologies Pte. Ltd., Singapore). The cycling conditions were set as follows: 1 cycle of 55 °C for 2 min, 42 °C for 60 min, and 94 °C for 2 min; 5 cycles of 94 °C for 30 s, 44 °C for 45 s and 68 °C for 3 min 30 s; and 45 cycles of 95 °C for 30 s, 57 °C for 45 s and 68 °C for 3 min 30 s, followed by an extension of 68 °C for 10 min. The PCR products were measured using the Qubit dsDNA HS Assay Kit (Thermo Fisher Scientific) and diluted to 1 ng/μL of DNA. The libraries were prepared using the Nextera XT DNA Library Preparation Kit (Illumina, Inc. San Diego, CA, USA) and the quality was checked using the Agilent 2100 Bioanalyzer (Agilent Technologies, Palo Alto, CA, USA). The pooled libraries were then run on an Illumina MiSeq (2 × 250 bp, San Diego, CA, USA). The short NGS reads were quality-checked using UGENE v39 [37] and adaptor sequences were trimmed using Trimmomatic v0.39 [38]. De novo assembly of the reads was performed using SPAdes genome assembler v3.13 [39] and the contigs were blasted using BLAST v2.2.30 [40] against a local influenza virus database downloaded from NCBI. The reads were then mapped to the reference influenza genome. We recovered complete genomes of four novel A(H5N8) viruses and the sequences were deposited in the NCBI GenBank database (see Table 1 for accession numbers).

### 2.3. Temporal Phylogenetic Analysis and Molecular Characterization

All available influenza A(H5Nx) sequences were downloaded from NCBI and GISAID databases (as of 4 March 2021). Large dataset phylogenies were reconstructed using FastTree in Geneious v7.1.9 (Biomatters Ltd., Auckland, New Zealand) and the datasets were then sub-sampled to include clade 2.3.4.4a–h viruses. For each gene segment, dated phylogenies and changes in relative genetic diversity were estimated using the uncorrelated lognormal relaxed clock with the Gaussian Markov random field (GMRF) Bayesian skyride model and the SRD06 codon position model in BEAST v1.10.4 [41]. At least four independent runs of 100 million generations were used. The convergence of runs was checked using Tracer v.1.7.1 [42] after excluding burn-in values to ensure the effective sampling size values for all parameters were >200. The runs were combined using LogCombiner and the resulting maximum clade creditability (MCC) trees were summarized using TreeAnnotator. Ancestral amino acid mutations at the nodes were determined for each gene segment phylogeny using the treesub program [43].

## 3. Results

### 3.1. Prevalence of AIV in Pakistani Live Bird Markets

During 2018–2019, we detected 77 (17.1%) of 449 samples from chickens and quails as being positive for influenza A virus based on hemagglutination inhibition assay and NGS sequencing (Table 2), including 4 with A(H5N8) virus and 73 with A(H9N2) virus. The positivity rate of influenza A virus in chickens was higher in two LBMs (31.6% in Tollinton and 31.7% in Shahdara) in the Lahore district compared to LBMs in Sheikhupura and Sharaqpur (4.9–13.0%). Quail samples could only be collected from Tollinton market and showed high proportions of influenza A virus (28.6%) (Table 2). We also tested 696 environmental samples, of which 6 (0.9%) were positive for influenza A virus. Four environmental samples were detected in Lahore LBMs: three samples from chopping boards and one sample from sewage water. Two environmental samples from Sheikhupura LBMs were also positive: one sample from cage drinking water and one sample from a meat weighing scale.

### 3.2. Evolutionary Relationships of Avian A(H5N8) Clade 2.3.4.4b Viruses

**Figure 2:**
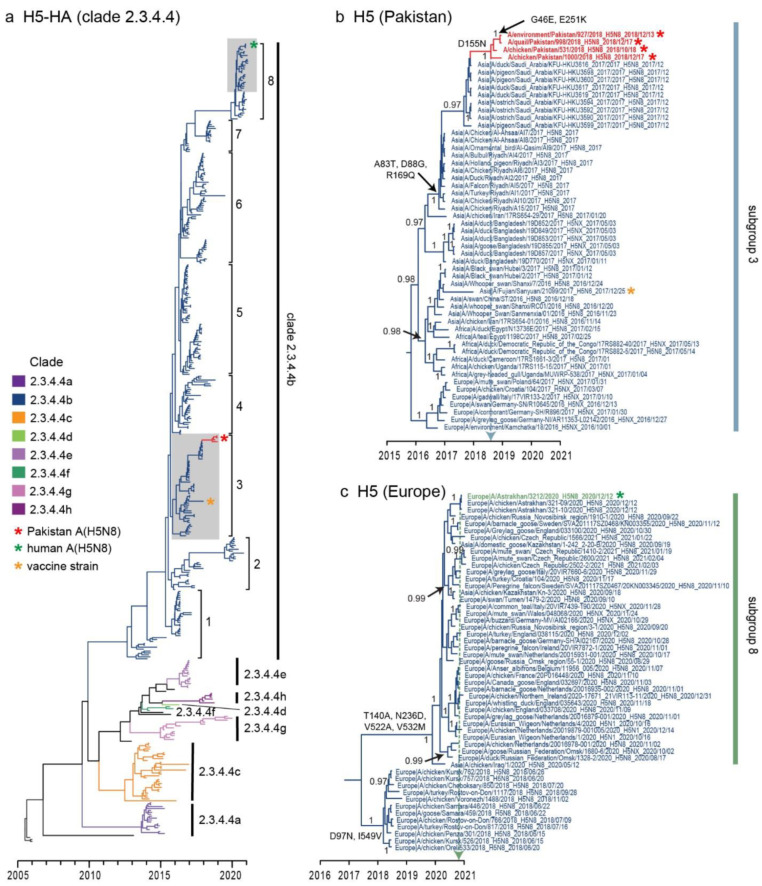
Evolutionary relationships of the H5-HA gene of highly pathogenic clade 2.3.4.4 viruses during 2005–2021. (**a**) Dated phylogeny of the H5-HA gene sequences. Branch colors represent eight different clades of 2.3.4.4 viruses (2.3.4.4a–2.3.4.4h). Eight subgroups of clade 2.3.4.4b are marked on the tree (subgroup 1–8). Grey shaded areas contain Pakistani (denoted by red asterisks) and human A(H5N8) viruses (denoted by green asterisk); the orange asterisk represents the vaccine strain for clade 2.3.4.4b virus. (**b**) Inset shows the H5-HA of novel Pakistan A(H5N8) viruses indicated by red asterisks. The dotted vertical line denotes the estimated mean time to the most common ancestor (TMRCA) for A(H5N8) viruses in Pakistan. (**c**) Inset shows the H5-HA phylogeny of human A(H5N8) virus from Russia. The dotted vertical line denotes the estimated mean time to the most common ancestor (TMRCA) for human A(H5N8) and closely related chicken A(H5N8) viruses in Russia. Amino acid mutations (H5 numbering) are indicated at major nodes. Bayesian posterior probability (PP) values greater than 0.95 are shown in panels (**b**,**c**).

From the 77 Pakistani influenza-A-positive samples, we recovered A(H5N8) virus genomes from 2 chickens, 1 quail and 1 environmental (chopping board) sample collected from Lahore LBMs (Table 1). Phylogenetic analysis of the H5-HA gene clearly showed that all four Pakistan A(H5N8) viruses belong to HPAI clade 2.3.4.4b viruses (Figure 2a). Clade 2.3.4.4b viruses began to diverge from other clade viruses from 2013 onwards, although subsequently underwent rapid diversification in 2017, with at least eight different subgroups now apparent (subgroups 1–8). These viruses are also widespread across different regions in Africa, East Asia, Europe, South Asia and the Middle East. All Pakistan H5 viruses shared a high level of nucleotide similarity (99.7–100%) and clustered within subgroup 3, forming a well-supported monophyletic lineage (Figure 2b, PP = 1).

The mean time of the most recent common ancestor (TMRCA) of all Pakistan H5-HA viruses is estimated as August 2018 (blue dotted arrow in Figure 2b; 95% HPD: May 2018–October 2018, Table 3), approximately three months prior to virus detection in domestic poultry. Pakistan A(H5N8) viruses are also most closely related to Saudi Arabian A(H5N8) viruses circulating in ducks, pigeons and ostriches, with a TMRCA of around November 2017 (95% HPD: October 2017–December 2017), suggesting movement of A(H5N8) from Saudi Arabia to Pakistan during November 2017–August 2018. Interestingly, subgroup 3 contains the recommended WHO candidate vaccine virus, A/Fujian-Sanyuan/21099/2017 (H5N6).

### 3.3. Molecular Characterizations of Pakistan A(H5N8) Viruses

**Figure 3:**
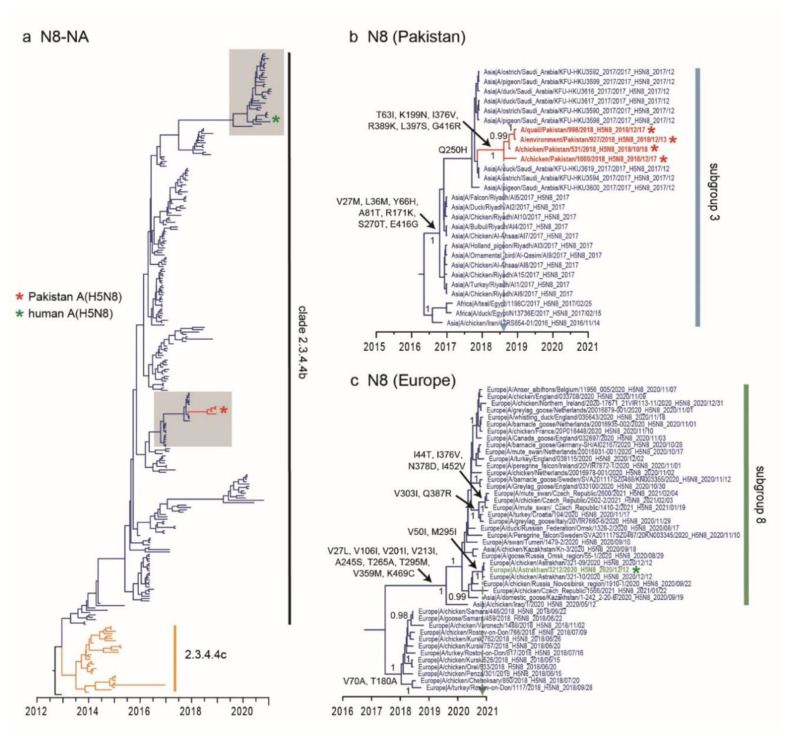
Evolutionary relationships of the N8-NA gene of highly pathogenic clade 2.3.4.4 viruses during 2005–2021. (**a**) Dated phylogeny of the N8-NA gene sequences. Grey shaded areas contain Pakistani and human A(H5N8) viruses. (**b**) Inset shows the N8-NA of novel Pakistan A(H5N8) viruses (denoted by red asterisks). The dotted vertical line denotes the estimated mean time to the most common ancestor (TMRCA) of A(H5N8) viruses in Pakistan. (**c**) Inset shows the N8-NA phylogeny of human A(H5N8) virus from Russia indicated by green asterisk. The dotted vertical line denotes the estimated mean time to the most common ancestor (TMRCA) of human A(H5N8) and closely related chicken A(H5N8) viruses in Russia. Amino acid mutations (N8 numbering) are indicated at major nodes. Bayesian posterior probability (PP) values greater than 0.95 are shown in panels (**b**,**c**).

The HA genes of all Pakistan A(H5N8) viruses possessed the polybasic amino acid cleavage site (PLREKRRKR/GLF), identical to other clade 2.3.4.4b viruses. Pakistan A(H5N8) viruses also have a D155N HA mutation (H5 numbering), with additional G46E and E251K mutations in 1 quail and 1 environmental sample. Mutations S155N and E251K in A(H5N1) virus are known to increase HA affinity to human α-2,6 receptors [44]. The N8-NA genes of Pakistan A(H5N8) viruses also fall within subgroup 3 (Figure 3a) and are most closely related to viruses from Saudi Arabia (Figure 3b). Consistent with H5-HA, the N8-NA genes of Pakistan viruses emerged around Aug 2018 (95% HPD: May 2018–October 2018, Table 3). We identified six NA mutations (T63I, K199N, I376V, R389K, L397S and G416R) among Pakistani viruses, as well as seven NA mutations among Saudi Arabian viruses (Figure 3b). Similarly, the N8-NA gene of human A(H5N8) and closely related chicken A(H5N8) viruses from Astrakhan were clustered within subgroup 8, which had acquired amino acid mutations (V50I and M295I) on the NA genes (Figure 3c).

The internal gene constellations of Pakistan A(H5N8) viruses are unequivocally derived from the same Saudi Arabia A(H5N8) lineage (Supplementary Figures S1–S8). The mean TMRCAs of the six internal gene segments fall in a similar period to the surface proteins, i.e., June 2018–September 2018 (Table 3), indicating introduction of A(H5N8) as a whole virus into LBM poultry in Pakistan. The internal gene segments of Pakistan A(H5N8) viruses and those from the closely related Saudi Arabia A(H5N8) lineage all possessed a number of significant non-synonymous mutations (Supplementary Figures S1–S3, S5, S7 and S8). Among these, PB2-I292V is associated with increased virus replication in mammalian cells [45,46], PB1-D622G and PA-V44I have been shown to increase polymerase activity and virulence of H5N1 virus in mice [44,47], PA-N321K is related to increased polymerase activity of pandemic H1N1 virus in human cells [48] and M2-S31N indicates amantadine resistance [49].

### 3.4. Diversity of Clade 2.3.4.4b Viruses Associated with Human Infection

**Figure 4:**
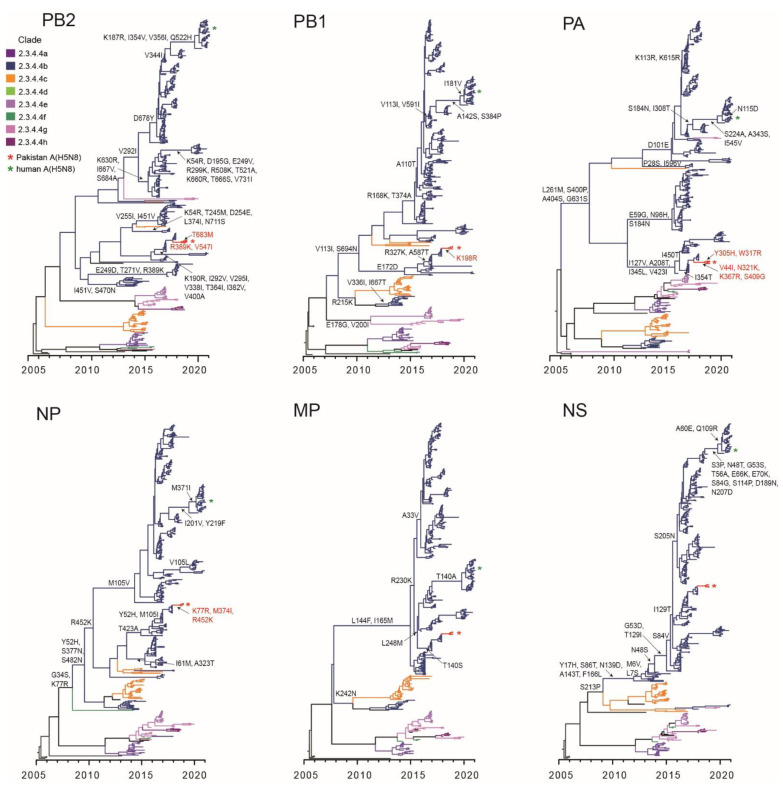
Evolutionary relationships between the internal genes of highly pathogenic clade 2.3.4.4 viruses during 2005–2021. Novel Pakistan A(H5N8) viruses are denoted by red asterisks, whereas the human A(H5N8) virus from Russia is indicated by green asterisks. Significant amino acid mutations are indicated at major nodes.

Early clade 2.3.4.4b viruses were detected in geese and ducks in China during 2013–2014. The mean TMRCA estimates of the HA and NA genes of clade 2.3.4.4b are August 2012 (95% HPD: October 2011–April 2013) and April 2013 (95% HPD: November 2012–August 2013), respectively. We also observed several key mutations of clade 2.3.4.4b that occurred on the trunk of phylogenetic trees (Figure 4). Some of these mutations are associated with increased virulence and transmission of avian viruses in mammals—PB2-D678Y mutation allows the transmission of A(H7N9) among ferrets [50], while PA-G631S and NP-M105V mutations have been shown to increase virulence of A(H5N1) virus in mice and chickens [51,52]. All clade 2.3.4.4 viruses, with the exception of clade 2.3.4.4b, possess an NS1 deletion at amino acid positions 80–84 [53,54] that may increase the virulence of A(H5N1) in chickens and mice [55].

**Figure 5:**
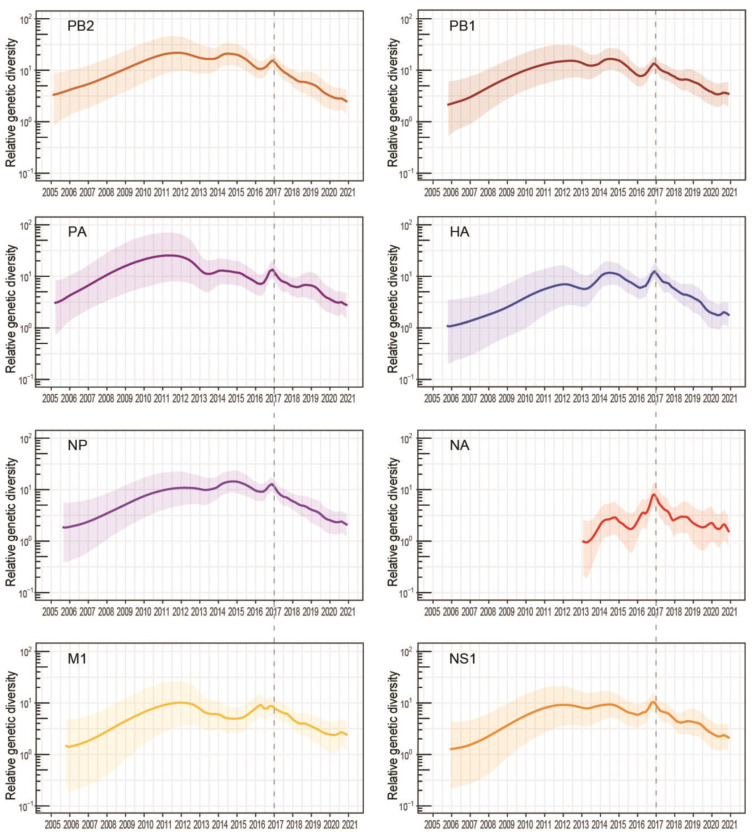
Population dynamics of each gene segment of highly pathogenic A(H5N8) clade 2.3.4.4 viruses during 2012–2021. The relative genetic diversity was estimated using the Gaussian Markov random field (GMRF) Bayesian skyride model in BEAST, based on 410 nucleotide sequences (except N8-NA with 275 nucleotide sequences). The dotted vertical lines indicate the increased diversification of clade 2.3.4.4 viruses in 2017.

Skyride analysis of all eight gene segments of clade 2.3.4.4b viruses exhibited an increase in genetic diversity that peaked in early 2017 (vertical dotted lines in Figure 5), which also coincided with the diversification of H5Nx clade 2.3.4.4 subgroups (Figure 2a). More significantly, clade 2.3.4.4b viruses have acquired non-synonymous mutations on most viral genes, particularly on the HA, NA, polymerase and NS genes (Figure 4, Supplementary Figures S1–S8), indicating that the increased genetic diversity of clade 2.3.4.4b viruses could arise through genetic drift among poultry populations.

The viruses from Pakistan are distantly related to A/Astrakhan/3212/2020, responsible for the first human A(H5N8) infection in Russia in late December 2020, clustering in subgroup 8 that contains clade 2.3.4.4b A(H5N8) viruses which circulated in domestic and wild birds across Europe during May 2020–February 2021 (Figure 2c). The mean estimated TMRCA of the HA-H5 virus of subgroup 8 was estimated in early December 2019 (95% HPD: May 2019–March 2020), consistent with previous observation [56]. The H5-HA phylogeny shows that the human strain (A/Astrakhan/3212/2020) is most closely related to two chicken A(H5N8) clade 2.3.4.4b viruses with an estimated TMRCA in late November 2020 (green dotted arrow in Figure 2c, 95% HPD: October 2020–December 2020), indicative of direct transmission from domestic poultry. These recent European A(H5N8) viruses have four HA amino acid mutations (T140A, N236D, V522A and V532M) and are divergent from previously circulating chicken viruses in Russia from 2018 (Figure 2c). Taken together, our results suggest that the acquisition of amino acid mutations with known biological function emerged during the evolution of HPAI A(H5N8) viruses, highlighting the risk of zoonotic transmission at the avian–human interface.

## 4. Discussion

Live bird markets (LBMs) are associated with avian influenza transmission and outbreaks, providing environmental sources for virus persistence [57] and amplification, thereby increasing the risk of zoonotic infection. The multispecies composition of LBMs also poses risks for virus mutation and adaptation to different hosts. Through active influenza surveillance of LBMs in Pakistan, we observed varying levels of AIV positivity in domestic poultry, with LBMs in Lahore district having greater AIV prevalence compared to the LBMs in Sheikhupura and Gujranwala districts. Previous surveillance studies have reported the prevalence of AIV in Lahore, where low pathogenic A(H9N2) viruses have frequently been identified in domestic and commercial poultry [27,28,30,31,58]. The circulation of A(H9N2) has led to the generation of novel genotypes, increasing the risks of AIV infection in poultry and related occupational workers such as butchers and vaccinators [29,59,60].

Between 2018 and 2019, we detected and identified four avian HPAI A(H5N8) viruses in chickens and quails from LBMs in Lahore. We also detected environmental contamination with A(H5N8) from the same markets. Our phylogenetic analyses of Pakistan A(H5N8) viruses showed close genetic and phylogenetic similarities, all belonging to clade 2.3.4.4b viruses, showing that LBMs play a significant role in transmission of the virus among domestic birds. We showed that all eight gene segments of Pakistan HPAI A(H5N8) viruses were most closely related to viruses from Saudi Arabia, suggesting they were most likely introduced to Pakistan through the movement of live poultry from neighboring regions, while the similar TMRCA dates across all eight genes indicate that the viruses are likely derived from a single avian source that was introduced approximately three months before virus detection. The presence of viruses on chopping boards suggests that environmental contamination may occur through slaughtering or processing of infected live poultry. A previous study in China indicated that the highest H7 positivity rates came from chopping boards [21]. The prevalence rates of contamination from Pakistani LBMs in this study are also comparable to those of Bangladeshi LBMs [25]. Whilst the A(H9N2) virus has been frequently documented in Pakistan, no H5 or H7 viruses have been reported in poultry, although low levels of H5 antibodies (6.9%) have been reported in backyard poultry collected in 2009 [58].

The emergence of HPAI A(H5N8) clade 2.3.4.4b viruses in Pakistan in chickens is likely associated with poultry production. In Pakistan, poultry production has been growing significantly since the 1960s, with an increase of 126% in total meat production and an increase of 71% in total egg production during 2000–2010, with the main chicken exports being made to neighboring regions, including Afghanistan, Iran and Turkey [59]. A study examining the phylogeographic dynamics of A(H9N2) in Asia highlighted the migration rates from Pakistan to a number of countries, including India, Iran, Israel, Saudi Arabia and the United Arab Emirates [15]. Their data also indicated that live poultry trade and production are important drivers causing the spatial spread of A(H9N2) virus. In addition to domestic poultry, the HAPI A(H5N8) virus has been reported to the World Organization for Animal Health (OIE) by the Pakistan National Laboratory due to H5-positive wild birds (including mallard and swan) and macaws being found in Lahore Zoo, although no genetic data are available from those viruses, meaning cannot rule out introduction via wild birds. More surveillance data is needed to understand the transmission of A(H5N8) among domestic and wild birds in Pakistan and neighboring regions.

In this study, we also showed that clade 2.3.4.4b viruses exhibited markedly rapid diversification that peaked in 2017, with the acquisition of non-synonymous amino acid mutations on most viral genes. Some of these mutations are associated with mammalian adaptation, suggesting domestic poultry and wild birds are playing a significant role in virus evolution and potential cross-species transmission. Environmental contamination in LBMs also provides a persistent source for poultry infection, and effective control strategies to mitigate environmental contamination with AIV in LBMs are needed. The recent infections of poultry workers from Russia and of gray seals [60] indicate a broader host range for A(H5N8) viruses, highlighting their pandemic potential. At present, A(H5N8) clade 2.3.4.4 viruses have evolved into at least 8 distinct lineages, suggesting A(H5N8) variants are emerging at an unprecedented rate and pose a public health concern [61,62]. No sustained human-to-human transmission of A(H5) has been observed; however, the continual evolution and dissemination of A(H5N8) virus across multiple countries highlights the need for active influenza surveillance in domestic poultry and wild birds to detect emerging genetic variants and to assess their pandemic potential.

## Figures and Tables

**Table 1:** Novel avian A(H5N8) viruses in live bird markets in Pakistan.

Viruses	Date of Isolation	Market	District	GenBank Accession Number							
				PB2	PB1	PA	HA	NP	NA	MP	NS
A/chicken/Pakistan/531/2018	18 October 2018	Tollinton	Lahore	MZ701928	MZ701929	MZ701930	MZ701931	MZ701932	MZ701933	MZ701934	MZ701935
A/environment/Pakistan/927/2018	13 December 2018	Shahdara	Lahore	MZ702454	MZ702455	MZ702456	MZ702457	MZ702458	MZ702459	MZ702460	MZ702461
A/quail/Pakistan/998/2018	17 December 2018	Tollinton	Lahore	MZ702116	MZ702117	MZ702118	MZ702119	MZ702120	MZ702121	MZ702122	MZ702123
A/chicken/Pakistan/1000/2018	17 December 2018	Tollinton	Lahore	MZ702232	MZ702233	MZ702234	MZ702235	MZ702236	MZ702237	MZ702238	MZ702239

**Table 2:** Poultry and environmental samples tested for influenza A virus in live bird markets in Pakistan ^a^.

Sampling Location	Lahore District				Sheikhupura District				Gujranwala District				
	Tollinton Market		Shahdara Market		Sheikhupura Market		Sharaqpur Market		Clock Tower Market		Sheranwala Bagh Market		
	No. of Samples Collected	Positive Samples (%)	No. of Samples Collected	Positive Samples (%)	No. of Samples Collected	Positive Samples (%)	No. of Samples Collected	Positive Samples (%)	No. of Samples Collected	Positive Samples (%)	No. of Samples Collected	Positive Samples (%)	Positive Samples /Total Samples (%)
Bird species													
Chickens	96	31 (32.3%)	41	13 (31.7%)	92	12 (13.0%)	57	6 (10.5%)	60	5 (8.3%)	82	4 (4.9%)	71/428 (16.6%)
Quail	21	6 (28.6%)	0	NA	0	NA	0	NA	0	NA	0	NA	6/21 (28.6%)
Subtotal	117	37 (31.6%)	41	13 (31.7%)	92	12 (13.0%)	57	6 (10.5%)	60	5 (8.3%)	82	4 (4.9%)	77/449 (17.1%)
Environmental samples													
Chopping board surface	30	1 (3.3%)	19	2 (10.5%)	13	0 (0.0%)	26	0 (0.0%)	26	0 (0.0%)	13	0 (0.0%)	3/127 (2.4%)
Cage drinking water	29	0 (0.0%)	8	0 (0.0%)	24	1 (4.2%)	6	0 (0.0%)	30	0 (0.0%)	20	0 (0.0%)	1/117 (0.9%)
Cages	45	0 (0.0%)	22	0 (0.0%)	36	0 (0.0%)	27	0 (0.0%)	20	0 (0.0%)	10	0 (0.0%)	0/160 (0.0%)
Weighing scale	22	0 (0.0%)	11	0 (0.0%)	16	0 (0.0%)	15	1 (6.7%)	15	0 (0.0%)	11	0 (0.0%)	1/90 (1.1%)
Sewage water	25	1 (4.0%)	9	0 (0.0%)	18	0 (0.0%)	8	0 (0.0%)	25	0 (0.0%)	19	0 (0.0%)	1/104 (0.9%)
Water for meat processing	13	0 (0.0%)	18	0 (0.0%)	17	0 (0.0%)	20	0 (0.0%)	21	0 (0.0%)	9	0 (0.0%)	0/98 (0.0%)
Subtotal	164	2 (1.2%)	87	2 (2.3%)	124	1 (0.8%)	102	1 (0.9%)	137	0 (0.0%)	82	0 (0.0%)	6/696 (0.9%)
**Total**	**281**	**39 (13.9%)**	**128**	**15 (11.7%)**	**216**	**13 (6.0%)**	**159**	**7 (4.4%)**	**197**	**5 (2.5%)**	**164**	**4 (2.4%)**	**83/1145 (7.2%)**

^a^ Influenza-A-positive samples were identified by hemaglutination inhibition assay and next-generation sequencing.

**Table 3:** Estimated times to the most recent common ancestor (TMRCA) for Pakistan A(H5N8) viruses.

	Time to the Most Recent Common Ancestor (TMRCA)		
Gene Segment	Mean	95% Lower HPD *	95% Upper HPD
PB2	2018.43 (June 2018)	2018.08 (Jan 2018)	2018.67 (September 2018)
PB1	2018.58 (August 2018)	2018.30 (April 2018)	2018.75 (October 2018)
PA	2018.48 (June 2018)	2018.13 (February 2018)	2018.70 (October 2018)
HA	2018.6 (August 2018)	2018.38 (May 2018)	2018.75 (October 2018)
NP	2018.67 (September 2018)	2018.49 (June 2018)	2018.79 (October 2018)
NA	2018.61 (August 2018)	2018.36 (May 2018)	2018.77 (October 2018)
MP	2018.59 (August 2018)	2018.37 (May 2018)	2018.79 (October 2018)
NS	2018.63 (August 2018)	2018.31 (April 2018)	2018.79 (October 2018)

* HPD: highest posterior density.

## Data Availability

All the avian influenza sequences generated as a part of this study are publicly available in GenBank, individual accession numbers are provided in Table 1.

## Supplementary Materials

The following are available online at https://www.mdpi.com/article/10.3390/v13081633/s1, Figure S1. Evolutionary relationships of the PB2 gene of highly pathogenic clade 2.3.4.4 A(H5) influenza viruses; Figure S2. Evolutionary relationships of the PB1 gene of highly pathogenic clade 2.3.4.4 A(H5) influenza viruses; Figure S3. Evolutionary relationships of the PA gene of highly pathogenic clade 2.3.4.4 A(H5) influenza viruses; Figure S4. Evolutionary relationships of the H5-HA gene of highly pathogenic clade 2.3.4.4 A(H5) influenza viruses; Figure S5. Evolutionary relationships of the NP gene of highly pathogenic clade 2.3.4.4 A(H5) influenza viruses; Figure S6. Evolutionary relationships of the N8–NA gene of highly pathogenic clade 2.3.4.4 A(H5) influenza viruses; Figure S7. Evolutionary relationships of the MP gene of highly pathogenic clade 2.3.4.4 A(H5) influenza viruses; Figure S8. Evolutionary relationships of the NS gene of highly pathogenic clade 2.3.4.4 A(H5) influenza viruses.
