# Supplementary material for: Genetic Characterization of Highly Pathogenic Avian Influenza A(H5N8) Virus in Pakistani Live Bird Markets Reveals Rapid Diversification of Clade 2.3.4.4b Viruses
Source: Viruses. 2021 Aug 18;13(8):1633. doi: 10.3390/v13081633 (PMC8402709; doi:10.3390/v13081633)
Supplement: Supplementary file 1 [file viruses-13-01633-s001.zip › viruses-1312237-SI.pdf]

viruses

Supplementary Materials

Genetic Characterization of Highly Pathogenic Avian Influenza A(H5N8) Virus in Pakistani Live Bird Markets Reveals Rapid Diversification of Clade 2.3.4.4b Viruses

Muzaffar Ali [1,2], Tahir Yaqub [2], Muhammad Furqan Shahid [2], Foong Ying Wong [1], Nadia Mukhtar [2], Muhammad Naeem [3], Pauline Lam [1], Jayanthi Jayakumar [1], Gavin J. D. Smith [1,4,5] and Yvonne C. F. Su [1,*]

[1] Programme in Emerging Infectious Diseases, Duke-NUS Medical School, 8 College Road, Singapore 169857, Singapore; muzaffar.ali@uvas.edu.pk (M.A.); foongying.wong@duke-nus.edu.sg (F.Y.W.); pauline.lam@duke-nus.edu.sg (P.L.); jayanthi.jayakumar@duke-nus.edu.sg (J.J.); gavin.smith@duke-nus.edu.sg (G.J.D.S.)
[2] Institute of Microbiology, University of Veterinary and Animal Sciences, Lahore 54600, Pakistan; tahiryaqub@uvas.edu.pk (T.Y.); furqan.shahid@uvas.edu.pk (M.F.S.); nadiamukhtar84@yahoo.com (N.M.)
[3] Institute of Pure and Applied Biology, Bahauddin Zakariya University, Multan 60800, Pakistan; dr_naeembzu@yahoo.com
[4] SingHealth Duke-NUS Global Health Institute, SingHealth Duke-NUS Academic Medical Centre, Singapore 169857, Singapore
[5] Duke Global Health Institute, Duke University, Durham, NC 27710, USA
* Correspondence: yvonne.su@duke-nus.edu.sg

Citation: Ali, M.; Yaqub, T.; Shahid, M.F.; Wong, F.Y.; Mukhtar, N.; Naeem, M.; Lam, P.; Jayakumar, J.; Smith G.J.D.; Su Y.C.F. Genetic Characterization of Highly Pathogenic Avian Influenza A(H5N8) Virus in Pakistan Live Bird Markets Reveals Rapid Diversification of Clade 2.3.4.4b Viruses. *Viruses* **2021**, *13*, x. https://doi.org/10.3390/xxxxx

Academic Editors: Mathilde Richard and Ayato Takada

Received: 8 July 2021
Accepted: 16 August 2021
Published: date

Publisher's Note: MDPI stays neutral with regard to jurisdictional claims in published maps and institutional affiliations.

Copyright: © 2021 by the authors. Licensee MDPI, Basel, Switzerland. This article is an open access article distributed under the terms and conditions of the Creative Commons Attribution (CC BY) license (http://creativecommons.org/licenses/by/4.0/).

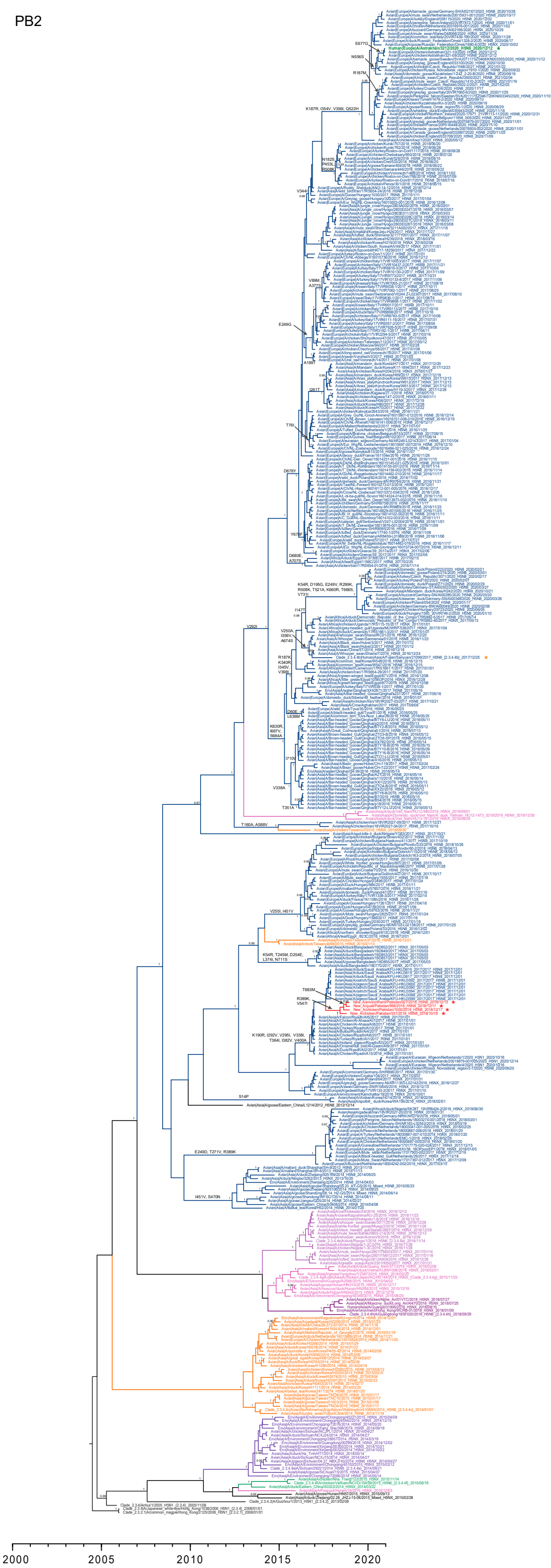

Figure S1. Evolutionary relationships of the PB2 gene of highly pathogenic clade 2.3.4.4 A(H5) influenza viruses. Branch colors represent eight different clades of2.3.4.4 viruses (2.3.4.4a–2.3.4.4h). Red asterisks indicate novel Pakistan A(H5N8)viruses; green asterisk indicates human A(H5N8) virus; and orange asterisk denotes vaccine strain. Significant amino acid mutations are indicated at major nodes. Bayesian posterior probability (PP) values greater than 0.95 are shown.

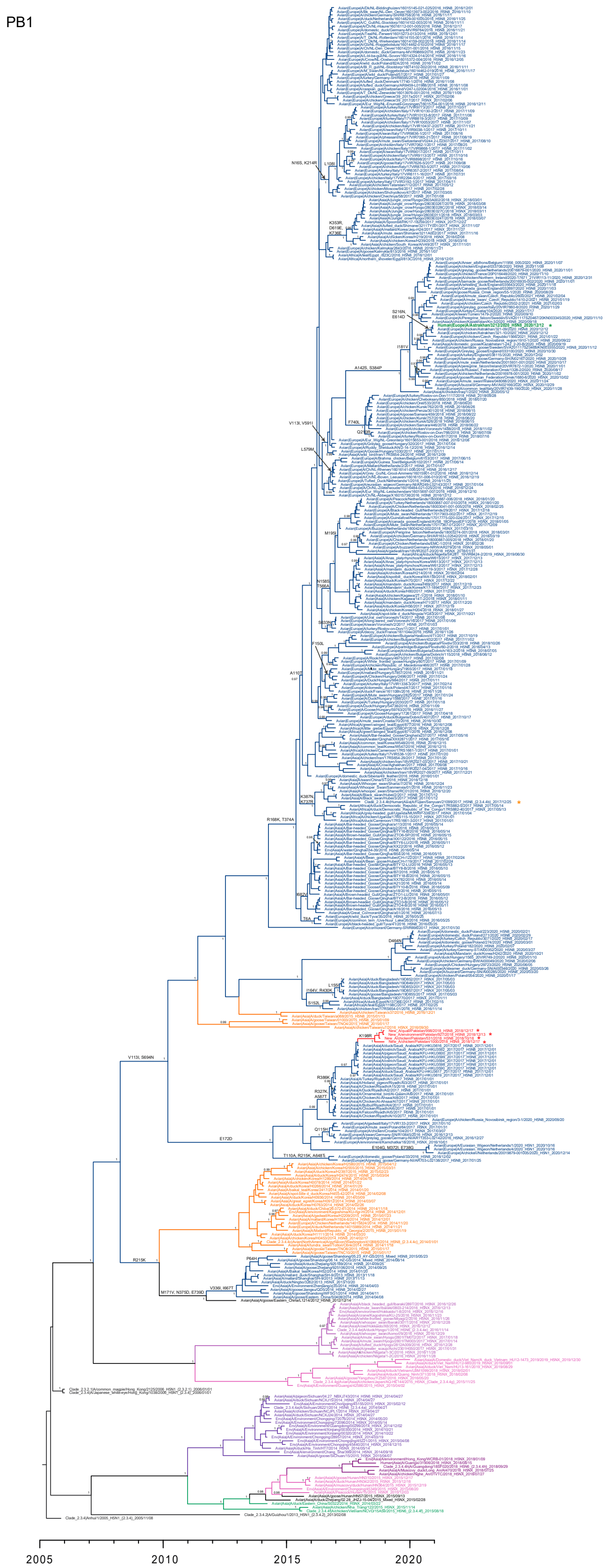

Figure S2. Evolutionary relationships of the PB1 gene of highly pathogenic clade 2.3.4.4 A(H5) influenza viruses. Branch colors represent eight different clades of 2.3.4.4 viruses (2.3.4.4a–2.3.4.4h). Red asterisks indicate novel Pakistan A(H5N8) viruses; green asterisk indicates human A(H5N8) virus; and orange asterisk denotes vaccine strain. Significant amino acid mutations are indicated at major nodes. Bayesian posterior probability (PP) values greater than 0.95 are shown.

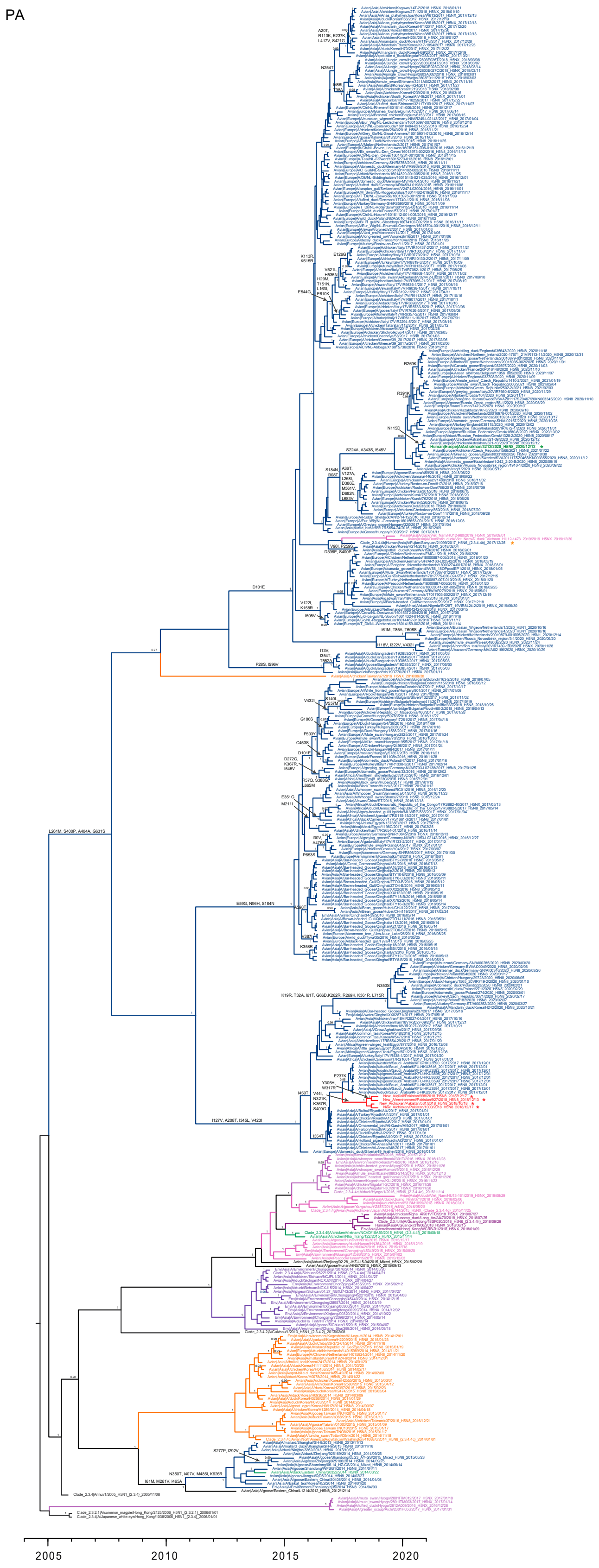

Figure S3. Evolutionary relationships of the PA gene of highly pathogenic clade 2.3.4.4 A(H5) influenza viruses. Branch colors represent eight different clades of 2.3.4.4 viruses (2.3.4.4a–2.3.4.4h). Red asterisks indicate novel Pakistan A(H5N8) viruses; green asterisk indicates human A(H5N8) virus; and orange asterisk denotes vaccine strain. Significant amino acid mutations are indicated at major nodes. Bayesian posterior probability (PP) values greater than 0.95 are show.

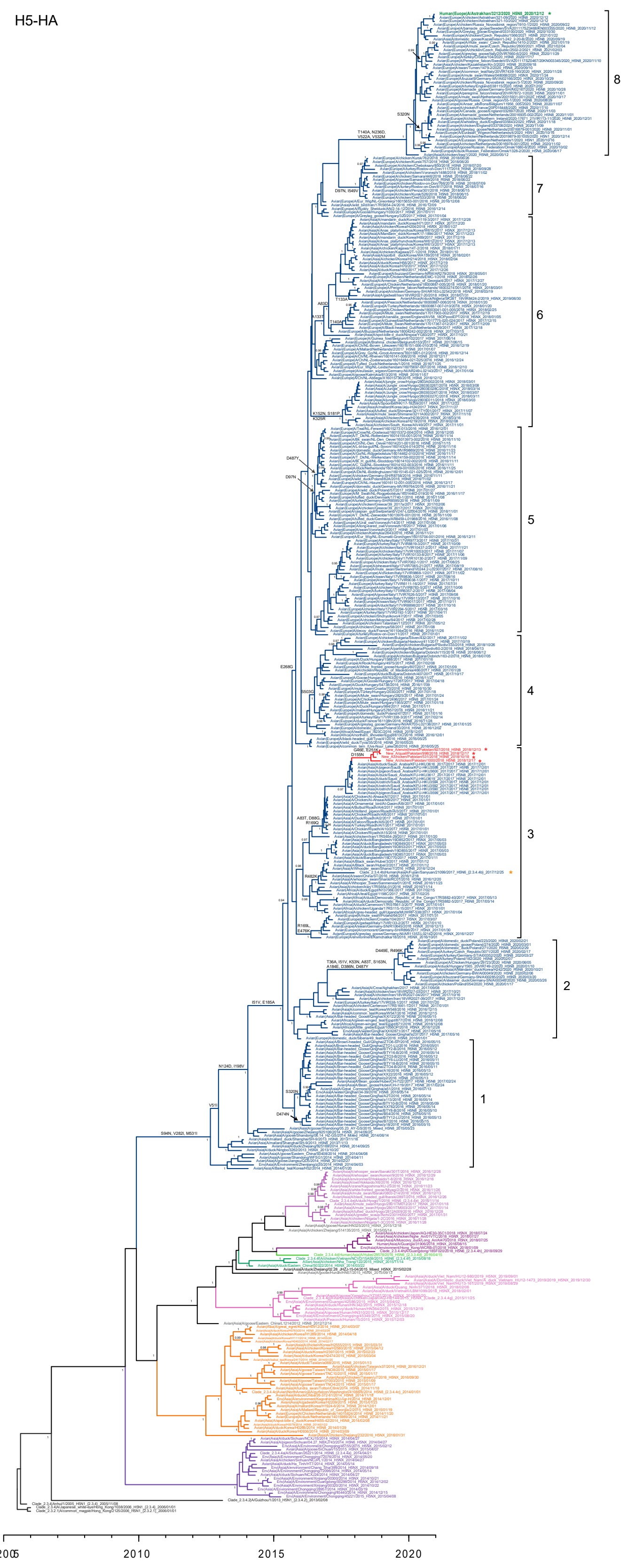

Figure S4. Evolutionary relationships of the H5-HA gene of highly pathogenic clade 2.3.4.4 A(H5) influenza viruses. Branch colors represent eight different clades of 2.3.4.4 viruses (2.3.4.4a–2.3.4.4h). Red asterisks indicate novel Pakistan A(H5N8) viruses; green asterisk indicates human A(H5N8) virus; and orange asterisk denotes vaccine strain. Significant amino acid mutations are indicated at major nodes. Bayesian posterior probability (PP) values greater than 0.95 are shown.

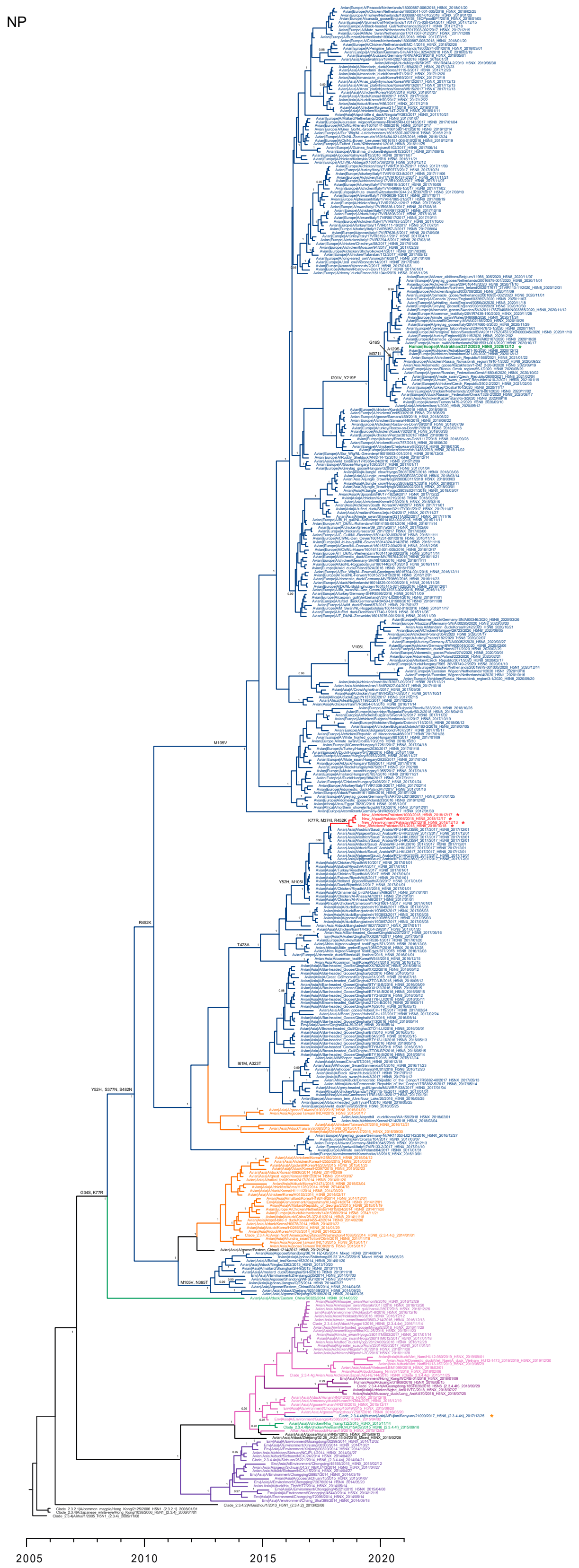

Figure S5. Evolutionary relationships of the NP gene of highly pathogenic clade 2.3.4.4 A(H5) influenza viruses. Branch colors represent eight different clades of 2.3.4.4 viruses (2.3.4.4a–2.3.4.4h). Red asterisks indicate novel Pakistan A(H5N8) viruses; green asterisk indicates human A(H5N8) virus; and orange asterisk denotes vaccine strain. Significant amino acid mutations are indicated at major nodes. Bayesian posterior probability (PP) values greater than 0.95 are shown.

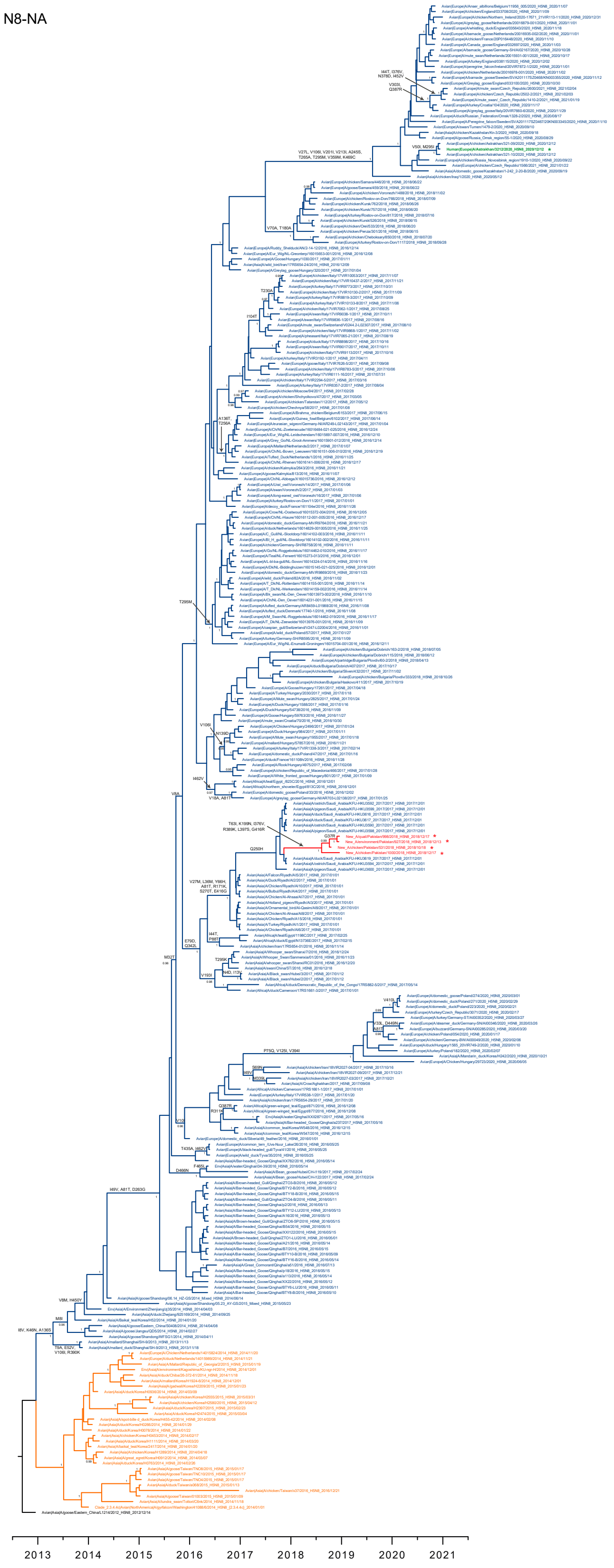

Figure S6. Evolutionary relationships of the N8–NA gene of highly pathogenic clade 2.3.4.4 A(H5) influenza viruses. Branch colors represent eight different clades of 2.3.4.4 viruses (2.3.4.4a–2.3.4.4h). Red asterisks indicate novel Pakistan A(H5N8) viruses; green asterisk indicates human A(H5N8) virus; and orange asterisk denotes vaccine strain. Significant amino acid mutations are indicated at major nodes. Bayesian posterior probability (PP) values greater than 0.95 are shown.

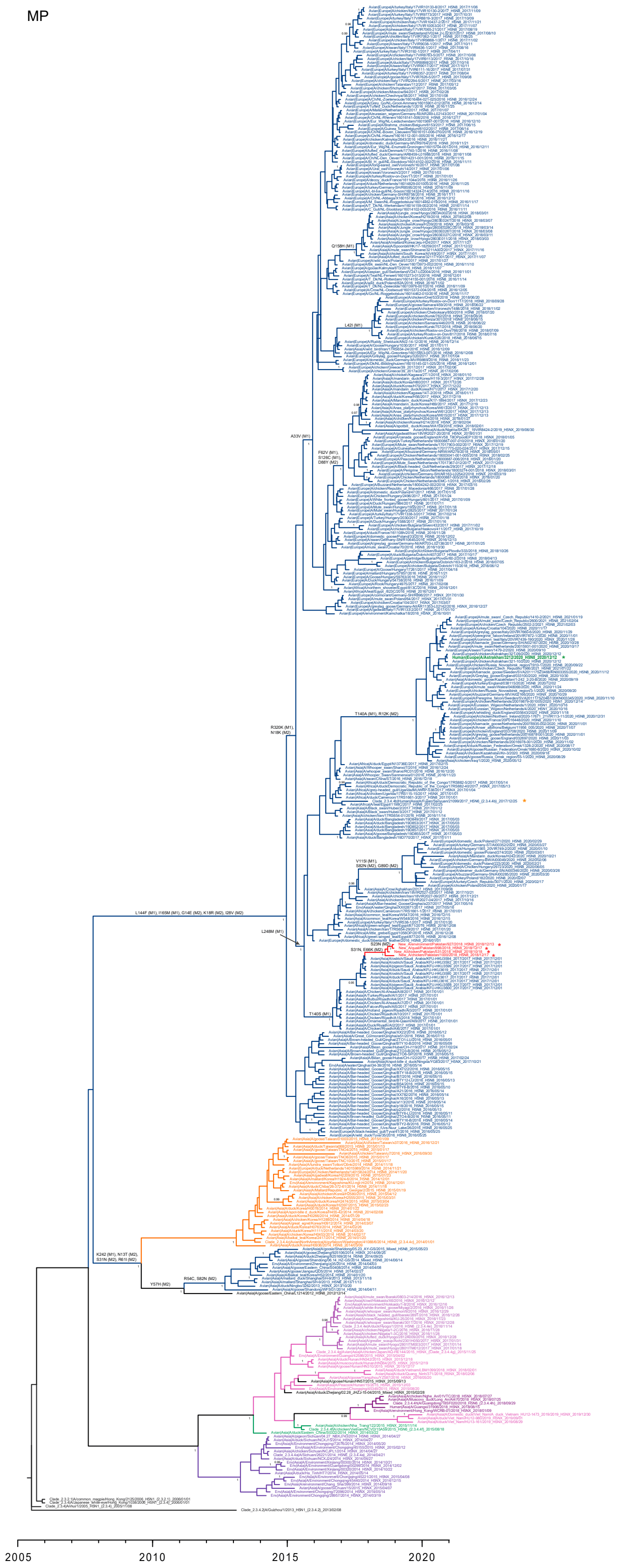

Figure S7. Evolutionary relationships of the MPgene of highly pathogenic clade 2.3.4.4 A(H5) influenza viruses. Branch colors represent eight different clades of 2.3.4.4 viruses (2.3.4.4a–2.3.4.4h). Red asterisks indicate novel Pakistan A(H5N8) viruses; green asterisk indicates human A(H5N8) virus; and orange asterisk denotes vaccine strain. Significant amino acid mutations are indicated at major nodes. Bayesian posterior probability (PP) values greater than 0.95 are shown.

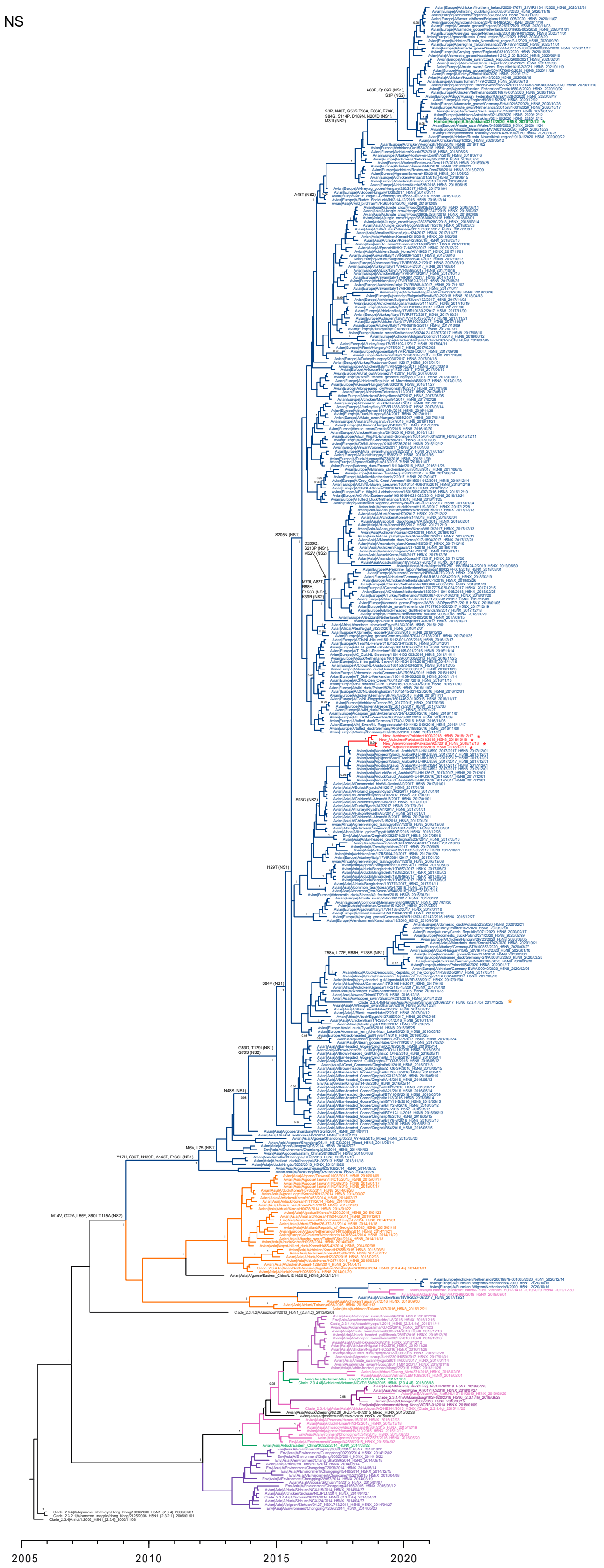

Figure S8. Evolutionary relationships of the NS gene of highly pathogenic clade 2.3.4.4 A(H5) influenza viruses. Branch colors represent eight different clades of 2.3.4.4 viruses (2.3.4.4a–2.3.4.4h). Red asterisks indicate novel Pakistan A(H5N8) viruses; green asterisk indicates human A(H5N8) virus; and orange asterisk denotes vaccine strain. Significant amino acid mutations are indicated at major nodes. Bayesian posterior probability (PP) values greater than 0.95 are shown.
